# Rapid Growth of TiO_2_ Nanoflowers via Low-Temperature Solution Process: Photovoltaic and Sensing Applications

**DOI:** 10.3390/ma12040566

**Published:** 2019-02-14

**Authors:** M. Shaheer Akhtar, Ahmad Umar, Swati Sood, InSung Jung, H. H. Hegazy, H. Algarni

**Affiliations:** 1 New and Renewable Energy Material Development Center (NewREC), Chonbuk National University, Chonbuk 54896, Korea; kjunggye@jbnu.ac.kr; 2 Department of Chemistry, Faculty of Science and Arts and Promising Centre for Sensors and Electronic Devices (PCSED), Najran University, Najran 11001, Saudi Arabia; 3 Department of Chemistry and Centre of Advanced Studies in Chemistry, Panjab University, Chandigarh 160014, India; swati17sood@gmail.com; 4 Department of Physics, Faculty of Science, King Khalid University; P.O. Box 9004, Abha 61421, Saudi Arabia; hosam_h_hegazy@yahoo.com (H.H.H.); halgarni@hotmail.com (H.A.); 5 Department of Physics, Faculty of Science, Al-Azhar University; Assiut 71524, Egypt

**Keywords:** TiO_2_ nanoflowers, photovoltaic device, chemical sensor, nitroaniline, sensitivity

## Abstract

This paper reports the rapid synthesis, characterization, and photovoltaic and sensing applications of TiO_2_ nanoflowers prepared by a facile low-temperature solution process. The morphological characterizations clearly reveal the high-density growth of a three-dimensional flower-shaped structure composed of small petal-like rods. The detailed properties confirmed that the synthesized nanoflowers exhibited high crystallinity with anatase phase and possessed an energy bandgap of 3.2 eV. The synthesized TiO_2_ nanoflowers were utilized as photo-anode and electron-mediating materials to fabricate dye-sensitized solar cell (DSSC) and liquid nitroaniline sensor applications. The fabricated DSSC demonstrated a moderate conversion efficiency of ~3.64% with a maximum incident photon to current efficiency (IPCE) of ~41% at 540 nm. The fabricated liquid nitroaniline sensor demonstrated a good sensitivity of ~268.9 μA mM^−1^ cm^−2^ with a low detection limit of 1.05 mM in a short response time of 10 s.

## 1. Introduction

Recently, metal oxide nanomaterials like titanium oxide (TiO_2_), zinc oxide (ZnO), nickel oxide (NiO), tin oxide (SnO_2_), cobalt oxide (Co_3_O_4_), iron oxide (Fe_2_O_3_), etc. have been regarded as some of the most important multi-functional materials because of their excellent functionalities and applications, such as electrochemical, photocatalytic, photo electrochemical, electronics, sensing, and so on [1,2,3,4,5]. Among various metal oxides, TiO_2_ nanostructures possess a special place because of their excellent properties, such as a wide band gap, low toxicity, easy synthesis, good stability, etc., and efficient applications in lithium ion batteries, solar cells, chemo-sensors, photocatalysts, and so on [5,6,7,8,9]. TiO_2_ nanostructures including nanotubes [10], nanoflakes [11], nanorods [12], solid and hollow spheres [13], and urchin-like [14] hierarchical TiO_2_ structures, have already been fabricated through a variety of solution techniques. In particular, the TiO_2_ nanostructures with 1D and 2D dimensions like nanorods, flowers, urchins, etc. often express additional extraordinary chemical/physical properties such as improved surface-to-volume ratio, fewer structural defects, and a large interspace [15,16]. M. Ghosh et al. reported on the fabrication of “nanorods-on-nanofiber” heterostructures by the distribution of V_2_O_5_ nanorods on TiO_2_ nanofibers via the gas jet fiber (GJF) spinning process, and these heterostructures showed excellent photocatalytic activity due to the slowdown of the electron–hole charge recombination phenomena [17]. H. Liu et al. recently studied the direct synthesis of TiO_2_ crystals with flower-like structures by a facile hydrothermal method using tetrabutyl titanate (TBT) as a titanium source and ethylene glycol as an additive [18]. They found that TiO_2_ crystals with flower-like structures exhibited high photocatalytic activity for the degradation of methylene blue (MB) under UV-vis light irradiation because these nanostructures had a considerably high surface area. Thus, it can be assumed that nanostructures with a flower-like structure might favor the achievement of high specific surface area and the enlargement of active sites. 

TiO_2_ is considered to be a pioneering material for dye-sensitized solar cells (DSSCs), which are photovoltaic devices that convert light energy to electrical energy through a semiconductor junction. Recently, the DSSC has become known as an emerging photovoltaic (PV) device owing to its low cost, simple manufacturing, and high solar-to-electrical conversion efficiency [19,20,21,22]. As a result, DSSCs are gaining wide research interests among the scientific community. Nanostructured TiO_2_ films have been used to fabricate DSSCs with light-to-electricity conversion efficiency (η) of 1.9% [23]. Using exposed mirror-like plane {001} facets of anatase TiO_2_, Zhang et al. fabricated efficient DSSCs as such mirror like facets exhibited light-reflecting ability, which led to better light to electricity conversion efficiencies [24]. Recently, Yang et al. fabricated hierarchical hollow microspheres of a TiO_2_-based DSSCs which possessed high conversion efficiency due to the specific and novel morphologies of the TiO_2_ material [25]. Thus, to obtain better photovoltaic performances, it is important to examine the various specific morphologies of TiO_2_. Herein, we report the photovoltaic performance of anatase-phase synthesized TiO_2_ nanoflowers by a facile low-temperature solution process. 

Along with photo-efficient energy conversion, metal oxides also find great applications in the sensing harmful and toxic gases, chemicals, or biomolecules. There is a great need for the efficient and sensitive detection of chemicals which are hazardous even in minimal concentrations, (e.g., toxic organic compounds like aliphatic and aromatic hydrocarbons, especially phenols, nitrophenols, nitroanilines, etc.). Recent studies have demonstrated that metal oxide nanomaterials can be used as efficient and sensitive chemo-sensors for volatile organic compounds (VOCs) such as ethanol, methanol, propanol, acetone, methylene chloride, benzene, butanol, xylene, isopropanol, and so on [26,27,28,29,30,31,32]. Among aromatic compounds, nitroaniline is considered a toxic chemical which is widely used in various pharmaceutical, dye, and pigment industries. However, the excessive use of nitroaniline and its persistence in the environment poses a serious threat to the health and wellness of living organisms. Thus, due to its highly stable and toxic nature, it is essential to monitor the presence of nitroaniline in the environment. Among various methods used to monitor the toxic chemicals, electrochemical-based sensors devised from metal oxide are considered to be an important class of sensors which provide an effective, efficient, and selective response [33,34,35,36]. Herein, the fabrication and characterization of anatase-phase TiO_2_ nanoflowers based on an efficient nitroaniline chemical sensor are reported. 

## 2. Materials and Methods

### 2.1. TiO_2_ Nanoflowers Synthesis

In a typical procedure, 0.04 M titanium chloride (TiCl_4_, Samchun Chemicals, Daejeon, Korea) was slowly added to 50 mL of deionized (DI) water in an ice bath and stirred for 10 min. After complete mixing of TiCl_4_, the reaction mixture was taken out of the ice bath and 50 mL DI water was added. A small amount (5 mL) of liquid ammonia (28% NH_3_, Samchun Chemicals, Daejeon, Korea) was mixed in the reaction mixture under continuous stirring, and was placed in the laboratory oven at 70 °C for 6 h which resulted in white precipitate. Consequently, the obtained precipitate was washed properly by ethanol and DI water. Later, the washed precipitate was dried in the oven at 60 °C for 24 h and calcined at 450 °C for 2 h. The final product was then characterized by different techniques to investigate the morphological, structural, compositional, optical, photovoltaic, and sensing properties. 

### 2.2. Characterizations of TiO_2_ Nanoflowers

The morphologies of the synthesized TiO_2_ nanoflowers were investigated using a field emission scanning electron microscope (FESEM, Hitachi S-4800, Tokyo, Japan), transmission electron microscope (TEM, JEM-ARM200F, JEOL, Peabody, MA, USA) and high-resolution (HR) TEM. The crystal and structural properties of the synthesized material were examined using X-ray diffraction (XRD, PANalyticalX’Pert PRO, Malvern Panalytical, Malvern, UK) and X-ray photoelectron spectroscopy (XPS, AXISNOVA CJ109, Kratos Inc., Manchester, UK). The XRD was measured in a range of 20–80° with Cu-Kα radiation (λ = 1.54178 Å). Energy-dispersive spectroscopy (EDS) connected with FESEM and Fourier transform infrared spectroscopy (FTIR, Nicolet, IR300, Thermo Fisher Scientific, Waltham, MA, USA) were used to observe the chemical and elemental compositions of the synthesized nanoflowers. The FTIR sample was prepared on KBr-based pellets and measured in the range 400–4000 cm^−1^. Room-temperature Raman-scattering (Raman microscope, Renishaw, UK) and UV–Vis spectroscopic techniques (2550 Shimadzu, Kyoto, Japan) were used to examine scattering and optical properties, respectively.

### 2.3. Fabrication and Characterization of DSSC Based on TiO_2_ Nanoflowers

The fabrication of a DSSC based on TiO_2_ nanoflowers was done as reported elsewhere [22]. For the photoanode, a slurry of the synthesized TiO_2_ nanoflower powder (0.2 g) was made by mixing it with aqueous polyethylene glycol (PEG, 4 wt.%) solution. An incremental addition of PEG solution was important to achieve a uniform TiO_2_ slurry. The prepared TiO_2_ slurry was spread by the doctor blade method over the framed fluorine-doped tin oxide (FTO) glass substrates and kept for 10 min to dry. Afterward, the TiO_2_-coated FTO substrate was calcined at 450 °C for 30 min. Consequently, the TiO_2_-coated FTO substrate was kept for dye absorption by immersing it into 0.3 mM ethanolic solution of N719 dye at room temperature for 16 h in the dark. After dye absorption of the required time, ethanol solvent was used to rinse the dye-absorbed TiO_2_-coated FTO substrate and removed non-absorbed dye from the TiO_2_ surface, and finally dried in an oven under nitrogen stream at 40 °C. To prepare the counter-electrode, a thin layer (~80 nm) of platinum (Pt) was coated with the ion sputtering technique on the cleaned FTO glass substrate. The DSSC assembly was made by combining the counter (Pt-coated FTO) and photoanode (dye-absorbed TiO_2_-coated FTO) and sealed with a separating Surlyn sealing sheet (60 μm) using a hot plate. The redox electrolyte, made of 0.05 mM I_2_, 0.2 M tert-butyl pyridine in acetonitrile, and 0.5 M LiI, was inserted through one of two small holes initially made on the counter electrode. The fabricated DSSC possessed an active area of 0.25 cm^2^. 

## 3. Results and Discussion

### 3.1. Crystalline and Structural Characterizations of TiO_2_ Nanoflowers

**Figure 1:**
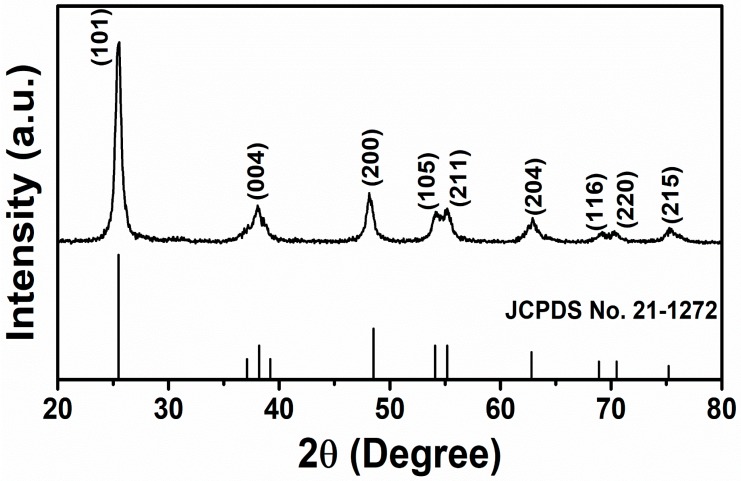
Typical XRD patterns of TiO_2_ nanoflowers synthesized by facile low-temperature solution process.

The crystalline properties of the synthesized TiO_2_ materials were examined by XRD analysis and the obtained diffraction peaks are demonstrated in Figure 1. 

The diffraction pattern of synthesized TiO_2_ constituted several well-defined peaks which were located at 2θ = 25.4°, 38.1°, 48°, 54.1°, 55.2°, 62.7°, 68.9°, 70.5°, and 75.2° and assigned as the lattice places of TiO_2_ (101), (004), (200), (105), (211), (204), (116), (220), and (215), respectively. The observed diffraction peaks were assigned to the typical TiO_2_ tetragonal crystal phase corresponding to anatase (JCPDS 21-1272) [37,38]. The crystallite size and full width half maxima (FWHMs) of synthesized TiO_2_ powders were accounted by Scherrer’s equation [39]. Using the diffraction peak at 25.4°, the synthesized TiO_2_ powders showed a small crystallite size of 12.23 nm and FWHM value of 0.7°. Other peaks corresponding to other crystalline forms or any other material were not observed, suggesting that the synthesized TiO_2_ was composed of pure anatase phase.

**Figure 2:**
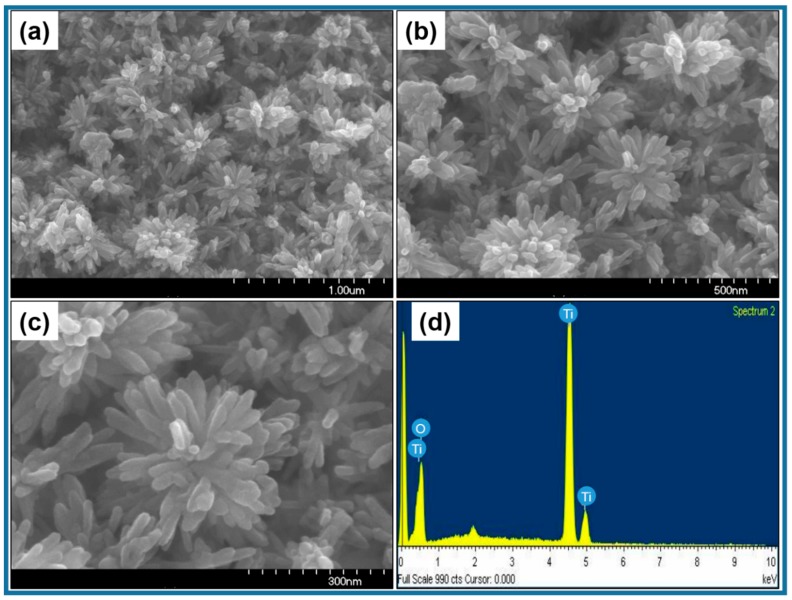
(**a**–**c**) FESEM images and (**d**) EDS spectrum of TiO_2_ nanoflowers synthesized by facile low-temperature solution process.

The morphology of synthesized TiO_2_ powders was investigated by performing the FESEM and TEM analysis. Figure 2a–c shows the FESEM images of the synthesized TiO_2_. At low magnification (Figure 2a,b), it could be well-observed that the synthesized TiO_2_ materials possessed a flower-like morphology and had grown in high density. The magnified FESEM image (Figure 2c) revealed that several rod-like structures with an average length of ~100 nm aggregated in three dimensions to form a flower-like morphology. The elemental composition of the synthesized TiO_2_ nanoflowers were observed from the EDS spectrum, as shown in Figure 2d. The prominent element peaks of Ti and O are seen in Figure 2d, which suggests that the synthesized materials were composed of only Ti and O. From quantitative analysis, the stoichiometric atomic weight % ratio of each element (83.69% for O and 16.31% for Ti) clearly confirms the formation of TiO_2_. No other peaks were seen for other any other element, suggesting the high purity of the TiO_2_ nanoflowers. 

**Figure 3:**
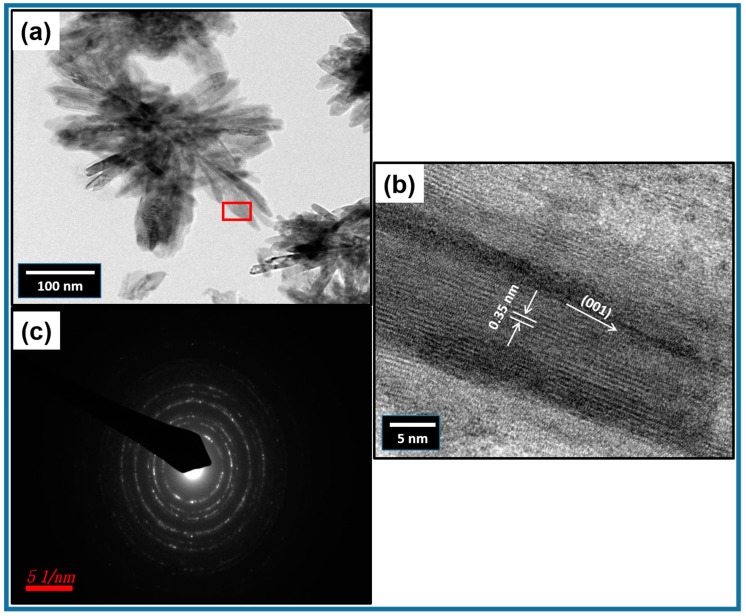
Typical (**a**) TEM, (**b**) HRTEM, and (**c**) SAED pattern of TiO_2_ nanoflowers synthesized by facile low-temperature solution process.

Synthesized TiO_2_ morphology was analyzed again by TEM and HRTEM analysis, as indicated in Figure 3. The low-resolution TEM image (Figure 3a) of the synthesized TiO_2_ clearly shows rods like petals which are aggregated to form a three-dimensional flower-like morphology. Flower-like TiO_2_ is in the nano-dimension, and it was estimated that each petal (rod) had an average size of ≤100 nm. Hence, the results of TEM were in accordance with the FESEM images. The HRTEM image (Figure 3b) presented well-defined lattice fringes with a difference in lattice spacing of 0.35 nm, that is promptly evidenced the typical lattice spacing of the (110) plane of anatase TiO_2_ [40,41]. Figure 3c depicts the selected area electron diffraction (SAED) patterns of as-synthesized TiO_2_ nanoflowers. SEAD patterns are the ring-like patterns which indicate the polycrystalline nature of TiO_2_. 

**Figure 4:**
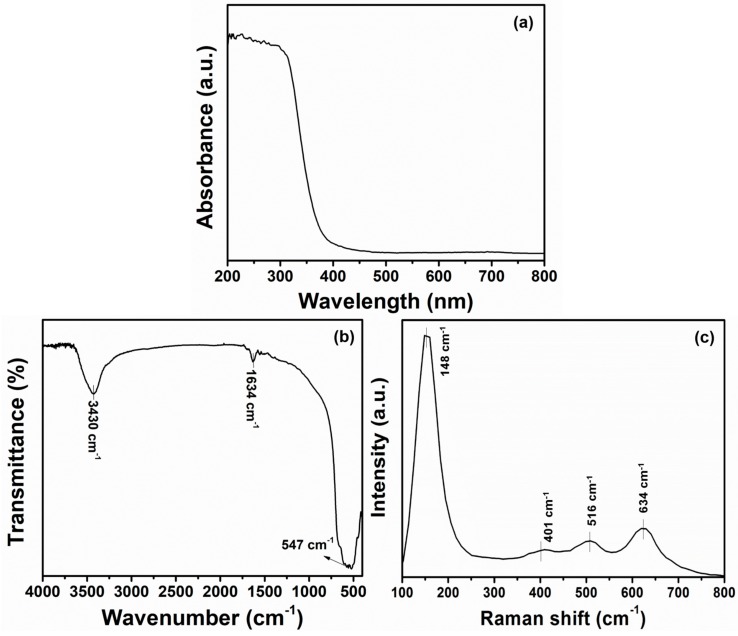
Typical (**a**) UV–Visible, (**b**) FTIR, and (**c**) Raman-scattering spectroscopy of synthesized TiO_2_ nanoflowers.

The optical properties of the synthesized TiO_2_ nanoflowers were investigated using UV–Vis spectroscopy, as indicated in Figure 4a. The synthesized TiO_2_ nanoflowers exhibited an absorption edge at 398 nm, which corresponded to an energy band gap of 3.2 eV. The obtained band gap value was well matched with the value of typical anatase TiO_2_ (i.e., E_g_ = 3.21 eV). Hence, from UV–Vis spectroscopic studies, it was again confirmed that the synthesized material was pure anatase TiO_2_. Moreover, the typical FTIR spectrum was also recorded in the range from 4000–400 cm^−1^ for the synthesized TiO_2_ nanoflowers to explain their structural properties. Figure 4b shows the synthesized TiO_2_ nanoflower FTIR spectrum. The peaks located at 3430 and 1634 cm^−1^ originated from the surface OH groups and H–O–H bending vibration of adsorbed water, respectively [42]. A very intense IR band located at 547 cm^−1^ was assigned to the Ti–O stretching in TiO_2_ lattice [42,43], which suggested the structure of TiO_2_. Raman scattering spectroscopy was used to further investigate the structural and scattering properties of the synthesized TiO_2_ nanoflowers in the range 100–800 cm^−1^ (Figure 4c). All the observed Raman bands in the range of 100–800 cm^−1^ corresponded to the representative peaks of O–Ti–O in TiO_2_ nano-materials [44]. The appearance of the Raman band at 148 cm^−1^ was indicative of anatase TiO_2_ [45]. Moreover, Raman bands at 634 cm^−1^ (E_g_ mode), 401 cm^−1^ (B_1g_), and 516 cm^−1^ (doublet of A_1g_ and B_1g_ modes) were associated to symmetric Ti–O vibration from the A_g_ symmetric modes of the TiO_6_ octahedra and the splitting of the degenerate mode of TiO_6_ octahedra [45]. The obtained results were typical for the anatase phase of TiO_2_ [44,45].

**Figure 5:**
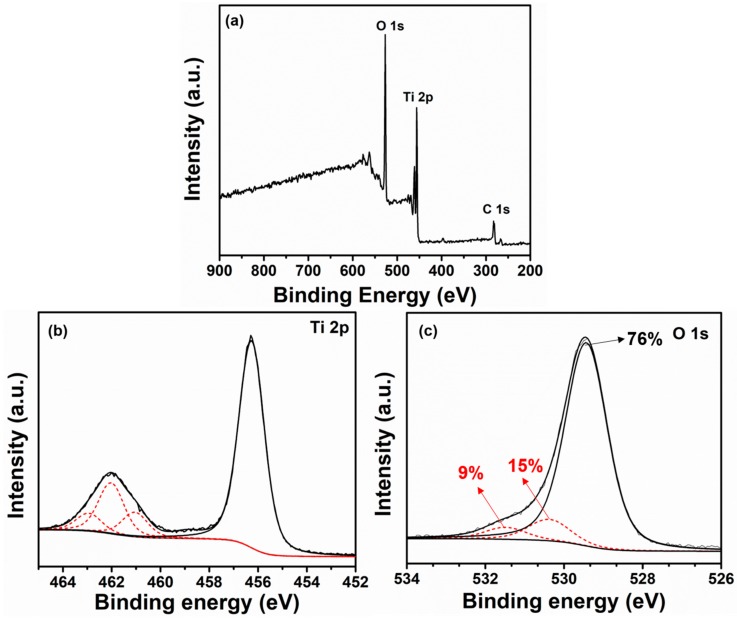
Typical XPS spectra of (**a**) survey, (**b**) deconvoluted high-resolution Ti 2p, and (**c**) deconvoluted high-resolution O 1s plots of as-synthesized TiO_2_ nanoflowers.

Further, to examine the surface composition, electronic environment, and oxidation states, XPS analysis was performed for the synthesized nanoflowers. As is known in the spectrum of the synthesized TiO_2_ nanoflowers, well-defined corresponding peaks of Ti, O, and C were obtained. A peak for C 1s at a binding energy of 284 eV was seen, attributable to the adventitious hydrocarbon from the XPS instrument. Figure 5b shows the core element XPS spectra for the Ti 2p binding energy region. The Ti 2p^3/2^ and Ti 2p^1/2^ spin-orbital splitting photoelectrons for the synthesized TiO_2_ nanoflowers specimen were seen at the binding energies of 456.8 and 462.3 eV, respectively. From the core element XPS spectrum for the O (1s) binding energy region (Figure 5c), it was observed that the peak corresponding to 529 eV corresponded to the lattice O of the TiO_2_ lattice. Two binding energies located at 530.3 and 531.5 eV originated from the adsorbed moisture (expressed by TiOH) and oxygen deficiencies or oxygen vacancies (expressed by TiOH) over the TiO_2_ surfaces. The existence of these defects (e.g., TiOH groups might helpful for the generation of oxygenated species such as hydroxyl ions, etc.) might take part in defining the sensing performance. With these observations, it is clear that the synthesized materials were again deduced to be anatase TiO_2_ nanoflowers.

**Figure 6:**
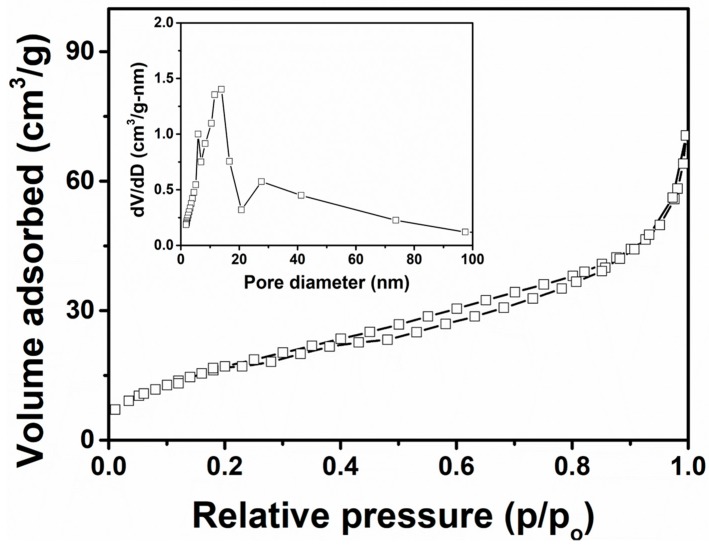
Nitrogen adsorption–desorption isotherms and pore-size distributions (inset) of as-synthesized TiO_2_ nanoflowers.

The as-synthesized TiO_2_ nanoflowers were further analyzed by measuring the N_2_ adsorption–desorption isotherms to elucidate the specific surface area and pore size distribution. As shown in Figure 6, the obtained isotherm displayed a hysteresis loop in the relative pressure ranging from 0.4–0.9. Isotherms were analyzed using Brunauer-Emmett-Teller (BET, Micromeritics Tristar 3000, Norcross, GA, USA) analysis, and as-synthesized TiO_2_ nanoflowers showed a specific surface area of ~63.34 m^2^/g. A pore size distribution plot is presented in the inset of Figure 6. It indicates that as-synthesized TiO_2_ nanoflowers developed mesopores, as observed by the distribution in the range of 5–20 nm. This result clearly displays the porous nature of as-synthesized TiO_2_ nanoflowers, which could be helpful for high dye absorption and the generation of large oxygenated species.

### 3.2. Characterizations of TiO_2_ Nanoflowers-Based DSSC

**Figure 7:**
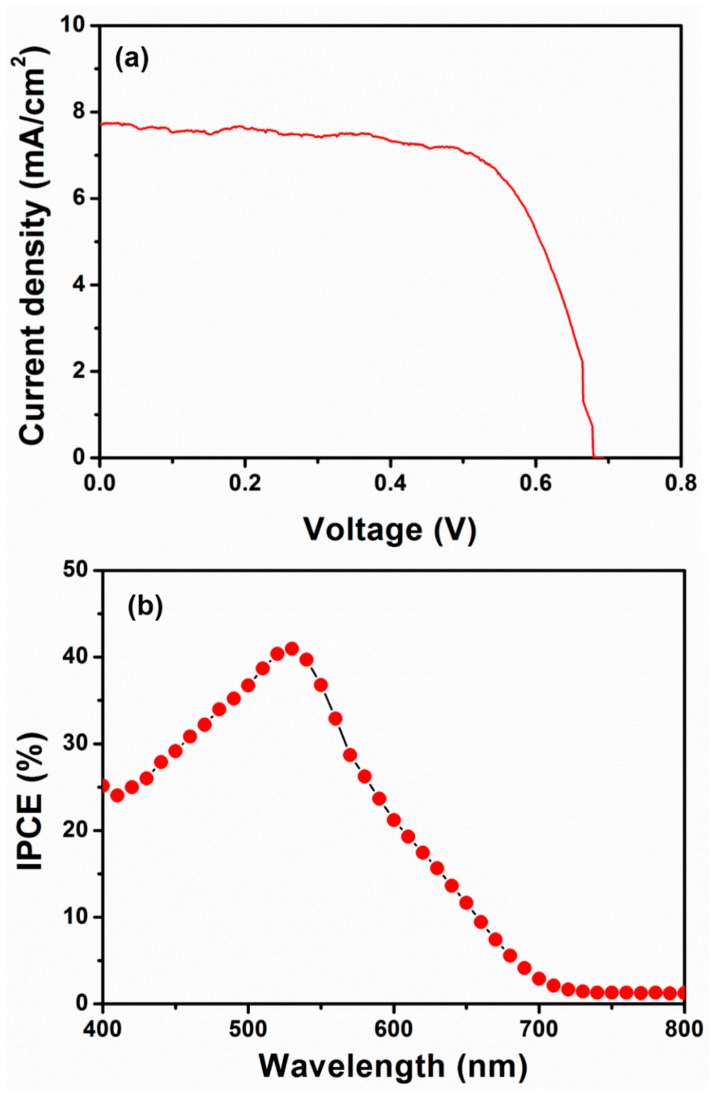
Typical (**a**) current–voltage (J–V) and (**b**) incident photon to current efficiency (IPCE) characteristics of TiO_2_ nanoflowers-based dye-sensitized solar cells.

In order to evaluate the photovoltaic properties of the synthesized TiO_2_ nanoflower-based photoanode, the photocurrent density (J)–voltage (V) of the fabricated DSSC was tested under 100 mW/cm^2^ light illumination (1 sun). Figure 7a shows the J–V curve of the fabricated DSSC with the synthesized TiO_2_ nanoflower-based photoanode. The fabricated DSSC yielded an overall conversion efficiency of ~3.64% along with a fill factor (FF) of ~0.67, open circuit voltage (V_OC_) of 0.693 V, and short circuit current density J_SC_ of ~7.80 mA cm^−2^. The high V_OC_ and FF of the device might be related to the generation of large grain sizes in the TiO_2_ nanoflower thin film, which resulted in the slightly high recombination rate at the interface of the TiO_2_ nanoflower/electrolyte. The low J_SC_ obtained by fabricated DSSC was associated with the low surface area of TiO_2_ nanoflowers (BET surface area = 63.34 m^2^/g), which might induce the light harvesting efficiency. To support low light harvesting efficiency and low J_SC_, an incident photon to current efficiency (IPCE) curve of the fabricated DSSC with synthesized TiO_2_ nanoflower based photoanode was investigated as a function of wavelength. Figure 7b depicts the IPCE curve of the fabricated DSSC with the synthesized TiO_2_ nanoflower-based photoanode in a wavelength range of 300–800 nm. The maximum IPCE was observed to be ~41% at 540 nm wavelength for the fabricated DSSC. From the IPCE curve, the estimated integrated J_SC_ value was 8.12 mA/cm^2^, which is in excellent agreement with the J_SC_ value obtained from the J–V curve. It was assumed that the TiO_2_ nanoflowers thin film poses a less-porous surface for dye loading, resulting in a low injection rate and high electron recombination. These factors might have retarded the photovoltaic performance and photocurrent density of the fabricated DSSC, and resulted in the low IPCE and low light harvesting efficiency. Zalas, M. et al. reported the fabrication of a DSSC based on TiO_2_ material which exhibited a photon-to-current efficiency of ~0.47% [46]. Similarly, Jang et al. demonstrated the use of an Al-incorporated TiO_2_ layer for a dye-sensitized solar cell application which exhibited an overall power conversion efficiency of ~2.81% [47]. Hu et al. presented the photo deposition of Ag nanoparticles on branched TiO_2_ nanorod arrays for dye-sensitized solar cells which presented light-to-electricity conversion efficiencies in the range of 1.87–2.83% [48]. Even though the performance was reasonable, the TiO_2_ nanoflower-based photoanode (in our case) showed higher photovoltaic performance when compared to similar photoanode based DSSCs in other scientific literature [44,46,47,48].

### 3.3. Properties of Nitroaniline Chemical Sensor Based on TiO_2_ Nanoflowers

**Figure 8:**
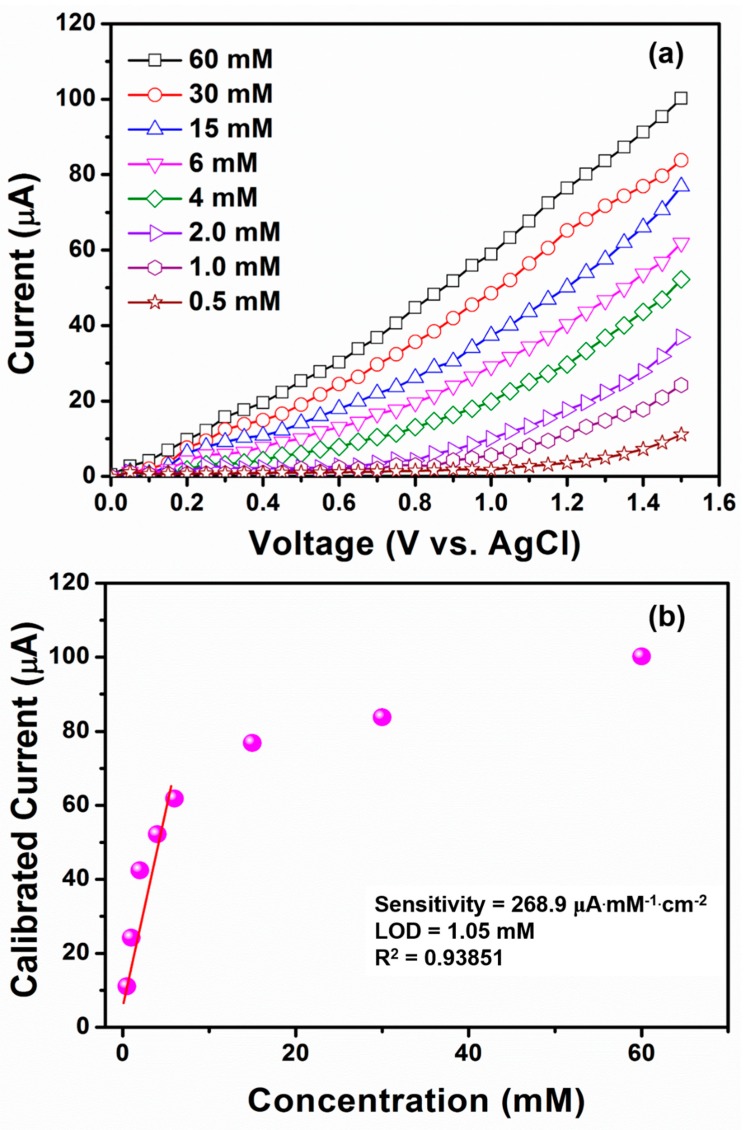
Typical (**a**) concentration-dependent (concentrations of 60–0.5 mM in 0.1 M PBS) I–V response of the fabricated TiO_2_ nanoflowers-based nitroaniline chemical sensor; (**b**) corresponding graph for calibration current vs. concentration of nitroaniline.

To fabricate the nitroaniline chemical sensor, a glassy carbon electrode (GCE) was modified with TiO_2_ nanoflowers which acted as an electron mediator material. The nitroaniline chemical sensor was designed in such a way that the TiO_2_ nanoflowers-modified GCE was used as a working electrode, while Pt wire acted as a counter-electrode in the phosphate buffer solution (PBS, 0.1 M). The effect of nitroaniline concentrations (0.5–60 mM) was studied by examining the I–V characteristics of several nitroaniline concentrations in PBS solution (pH = 7) at room temperature (298 K) with a relative humidity of 28%. From Figure 8a, the TiO_2_-nanoflower modified GCE exhibited a quick current response after the addition of a very low nitroaniline concentration in 1 M PBS, which clearly suggested a good electrochemical response towards nitroaniline. A series of I–V curves were performed with the TiO_2_ nanoflower-modified GCE with the different nitroaniline concentrations in 1 M PBS using the applied potential range from 0–1.5 V. As indicated in Figure 8a, with the increase in nitroaniline concentration, there was an increase in current response that could be attributed to the solution ionic strength improvement. This phenomenon proves very promising to increase the conductivity of chemo-sensors based on TiO_2_ nanoflowers. For detailed sensing parameters, the calibration plot for current vs. nitroaniline concentration was drawn from Figure 8a, as shown in Figure 8b. The current was linearly increased up to 6 mM afterward, and had a saturation level, which indicated the non-availability of ions at a higher nitroaniline concentration. Importantly, the chemo-sensor based on TiO_2_ nanoflowers expressed good linearity from 0.5–6 mM. A relatively high sensitivity of 268.9 μA mM^−1^ cm^−2^ along with a detection limit as low as 1.05 mM and response time (10 s) were attained by the fabricated nitroaniline sensor with a TiO_2_ nanoflowers-modified GCE. The good sensitivity and linearity over the TiO_2_ nanoflowers-modified GCE for nitroaniline sensing may be correlated with the specific flower morphology, which possessed superior surface-to-volume ratio, and thus exhibited very good adsorption and better electro-catalytic activities. The good sensing behavior of TiO_2_ nanoflowers-modified GCE can further be explained by understanding the sensing mechanism over the electrode surface. 

**Figure 9:**
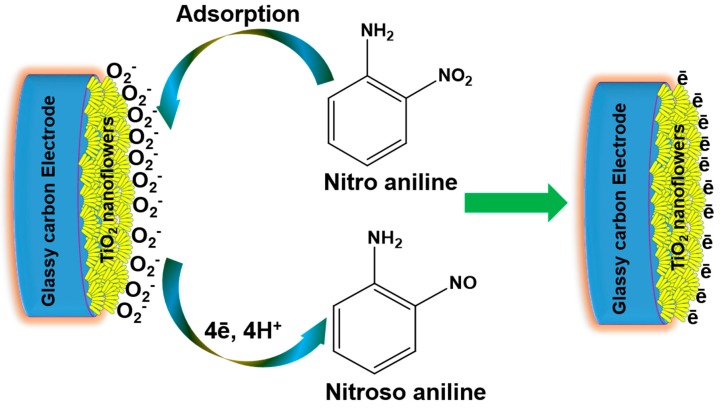
Illustration of possible nitroaniline chemical sensing over TiO_2_ nanoflowers-modified glassy carbon electrode (GCE).

Figure 9 shows the illustration of a possible mechanism for sensing response toward nitroaniline over the TiO_2_ nanoflowers-modified GCE. In the beginning of the sensing process, the TiO_2_ nanoflowers-modified GCE was primarily socialized with atmospheric oxygen molecules by taking an electron from the conduction band of TiO_2_. This process produced numerous active oxygen species, such as HO^−^, O^−^, O_2_^−^, etc. [49], as presented in Figure 9. These generated oxygen species were responsible for the chemisorption of nitroaniline chemical over the surface of the TiO_2_ nanoflowers-modified GCE. After interaction with the electrode surface, the nitroaniline chemical immediately oxidized into nitrosoaniline by leaving the electron on the surface of the TiO_2_ nanoflowers-modified GCE. Thus, the sensing properties of the TiO_2_ nanoflowers-modified GCE-based sensor was basically based on the good surface and electronic properties of TiO_2_ and the redox reactions that took place during the electrochemical measurement.

## 4. Conclusions

In summary, TiO_2_ nanostructures with flowerlike morphology and high crystallinity were synthesized using a facile chemical precipitation method. It was found that petal-like rods aggregated to form three-dimensional flower-like structures, grown in high density. In order to study the synthesized material surface composition, XPS studies were conducted and it was confirmed that highly pure TiO_2_ composed of Ti and O was prepared. The UV–Vis spectroscopic studies confirmed the preparation of anatase TiO_2_ with λ_max_ 398 nm, corresponding to an energy band gap of 3.2 eV. The synthesized TiO_2_ nanoflowers were used in the fabrication of a DSSC as photoanode materials, and achieved an overall efficiency of 3.64% with a maximum IPCE of ~41% at 540 nm wavelength. The synthesized TiO_2_ nanoflowers were also used for the electrochemical sensing of nitroaniline. The fabricated nitroaniline sensor with a TiO_2_ nanoflowers-modified GCE exhibited a sensitivity of 268.9 μA mM^−1^ cm^−2^ and a detection limit as low as 1.05 mM with a short response time. From this study, it is confirmed that the reported synthesis route is a facile method for the preparation of TiO_2_ with flower-like morphology and high crystallinity, which has great prospects in DSSCs and electro-chemical sensors.
